# Phenotypic Switching of VSMCs in the Development of CVDs: Focus on miRs

**DOI:** 10.3390/ijms26189078

**Published:** 2025-09-18

**Authors:** Ulyana Khovantseva, Yuliya Markina, Tatiana Kirichenko, Karina Goncharova, Diana Kiseleva, Vadim Cherednichenko, Alexander Markin

**Affiliations:** 1 Petrovsky National Research Center of Surgery, 119435 Moscow, Russia; 2 Department of Biology and Genetics, Petrovsky Medical University, 119435 Moscow, Russia; 3 Chazov National Medical Research Center of Cardiology, 121552 Moscow, Russia; 4 Faculty of Biology, Department of Biophysics, Lomonosov Moscow State University, 119991 Moscow, Russia

**Keywords:** cardiovascular diseases, microRNAs, phenotypic switching, vascular smooth muscle cells

## Abstract

Vascular smooth muscle cells (VSMCs) are one of the main cell types that constitute the vascular wall and play an important role in the development of cardiovascular diseases (CVDs). Normally, VSMCs are primarily involved in regulating vascular tone. Under pathological conditions, their phenotypic switching from a contractile to a pathological phenotype occurs, which is accompanied by impaired contractile function. These disorders lead to the development of different CVDs. In this regard, the aim of this review was to assess the influence of the features of differentiation pathways of VSMCs from embryonic progenitor cells and various transcription factors on the development of CVDs. Primary attention is given to the mechanisms of phenotypic switching of VSMCs, which plays a key role in the pathogenesis of vascular disorders. Particular emphasis is placed on investigating the effect of microRNAs (miRs) on the functional state of VSMCs. The analysis of current data demonstrates that alterations in miR expression result in significant functional rearrangements of VSMCs, which can contribute to the progression of CVDs. Consequently, understanding the mechanisms of VSMC dysfunction presents promising opportunities for developing targeted therapies aimed at restoring normal vascular wall function and preventing the development of CVDs.

## 1. Introduction

Vascular smooth muscle cells (VSMCs), which are involved in the development of cardiovascular diseases (CVDs) along with other cellular elements, play a significant role in their pathogenesis. The main functions of VSMCs include maintaining the structural integrity of the vessel wall, as well as the ability to contract and relax, through which blood flow is modulated. Disruption of these functions can lead to the development of CVDs, such as aneurysms and atherosclerosis, which occupy leading positions among the causes of disability and mortality in the world [1]. More than 500,000 people worldwide die from these diseases every year [2,3].

Atherosclerosis is a chronic inflammatory disease characterized by the formation of lipid plaques within the vessel wall [4]. With the progression of the disease, functional disorders of VSMCs occur, leading to hyperplasia, impaired contractile function, and alterations in synthetic activity of VSMCs [1]. The progression of atherosclerosis can lead to narrowing of the vessel lumen, resulting in blood clot formation, which may cause myocardial infarction or stroke. Despite significant therapeutic advances, atherosclerosis remains the leading cause of mortality worldwide [5]. Factors contributing to the development of atherosclerosis include high blood pressure, high cholesterol levels, smoking, and chronic inflammatory diseases such as arthritis, systemic lupus, or psoriasis [6].

An aortic aneurysm is characterized by localized dilation of the vessel wall, leading to its thinning, degradation of the extracellular matrix (ECM), reduction in the number of VSMCs, and subsequent wall rupture, which can result in fatal outcomes for the patient. The mortality rate in patients with ruptured aortic aneurysm ranges from 65% to 85% [7]. VSMC dysfunction plays an important role in the development of aneurysms, which may be caused by various risk factors: smoking, alcohol consumption, poor nutrition, genetic predisposition, and aging processes. It is noteworthy that this disease is more common in men, which might be due to the peculiarities of VSMC regulation under hormonal influence [8].

A healthy aortic wall consists of three layers: the innermost tunica intima, the middle tunica media, and the outermost tunica adventitia. Each layer is a complex system of connective tissue fibers, cells of mesenchymal origin, and elements of the *nervi vasorum* in the outermost layer [9]. The aortic layers include various cell types, such as VSMCs, fibroblasts, endothelial cells, multipotent stromal cells, vasa vasorum pericytes, etc. [10].

Normally, VSMCs are located in the middle layer of the aorta, where they actively synthesize ECM, have the ability to contract, and are involved in regulating blood pressure. During the development of aortic aneurysm, apoptosis, migration into the innermost layer of the aorta, and/or phenotype switching of VSMCs result in the reduction in ECM, which leads to a decrease in the strength and thickness of the vessel [11]. To date, it is believed that endothelial dysfunction is the main cause leading to atherosclerosis; however, in recent studies, scientists say that the phenotypic switching of VSMCs might also be an important factor in atherogenesis [12,13].

It is also worth noting that the process of CVD development can presumably depend on the origin of VSMCs. It is known that VSMCs are heterogeneous and have several sources of origin: the second heart field (SHF) [14], the neural crest, the somites, and the mesoderm [15]. Numerous studies have shown that depending on the embryonic origin, VSMCs can perform various functions in the development of CVDs, including aortic aneurysms [16,17].

Thus, understanding the regulatory mechanisms that control the differentiation of VSMCs from vascular precursors and the mechanisms of their phenotypic switching is important for studying their role in the development of CVDs and, as a result, developing therapeutic strategies for potential clinical use. The review examines publications investigating the origin of VSMCs, as well as factors involved in the process of VSMCs phenotypic switching and discusses potential mechanisms leading to the development of atherosclerosis and aortic aneurysm. The search for publications was based on the analysis of articles including the keywords “vascular smooth muscle cells, microRNA, cardiovascular diseases, phenotypic modulation” in the PubMed and Scopus databases up to July 2025.

## 2. Origin and Distribution of VSMCs

During embryonic development, angiogenesis occurs after the formation of the main vessels, during which the vessels differentiate into arteries or veins. The differentiation of VSMCs is a crucial stage in the process of angiogenesis, as they form the middle layer of arteries and veins. Embryonic VSMCs actively proliferate, migrate, and synthesize ECM proteins during differentiation [18].

The embryonic development of VSMCs is a complex process involving multiple regulatory signaling pathways and a mosaic pattern of differentiation, which is recognized as evolutionarily conserved across many different vertebrate species.

As mentioned above, VSMCs can originate from the neural crest and the SHF. The neural crest comprises mesenchymal cells and ectoderm. Neural crest cells represent a pluripotent cell population of the vertebrate embryo that originates in the neural folds. Cranial neural crest cells differ from trunk cells in their ability to undergo mesenchymal differentiation, generating a wide variety of mesenchymal tissues, such as the skeleton and connective tissue of the face, pericytes and VSMCs, meninges, dental papillae, and cornea [19]. The mesenchymal cells of the ectoderm migrate to the pharyngeal arches and excretory tract. Studies using chimeric chickens have demonstrated that neural crest cells can differentiate into VSMCs in thoracic arteries, as well as in vessels of the head and neck. Furthermore, it has been established that neural crest cells represent the sole cell lineage responsible for forming the smooth muscle of the arteries in the pharyngeal arches [20]. In subsequent studies, embryologists hypothesized that neural crest cells located at various levels of rhombomeres migrate into the arteries of the pharyngeal arches and differentiate into VSMCs on the third day of embryonic development in chickens [21,22,23]. These hypotheses were validated in a study where researchers demonstrated that dorsal and ventral cells originating from the neuroectoderm, but not from the mesoderm, are capable of differentiating into VSMCs of embryonic cerebral blood vessels [24]. However, the mechanisms of differentiation of VSMCs from the neural crest have not been fully studied to date [25]. Moreover, it has been demonstrated that the differentiation process can be influenced by environmental factors during specific periods of embryonic development. Probably, transforming growth factor-β (TGF-β) plays an important role in the differentiation process [26]. It is also worth noting that with the help of fate mapping, it was shown that the VSMCs originating from the neural crest are distributed throughout the aortic arch [27].

The SHF originates from the mesoderm, which constitutes part of the cardiac crescent and subsequently migrates into the developing cardiac tube. A subset of cells derived from the SHF within the heart tube forms the proximal segment of the thoracic aorta. Fate mapping studies have demonstrated that SHF-derived cells are localized in the aortic root and ascending thoracic aorta, while they do not contribute to the aortic arch [28].

Thus, the thoracic aorta contains overlapping VSMCs from both the neural crest and the SHF, and these sources exhibit a different distribution [29]. Current evidence suggests that the origin of VSMCs may significantly influence the progression of CVDs, including aneurysms and atherosclerosis. One of the studies demonstrated that the development of atherosclerotic lesions in response to inflammatory stimuli exhibited distinct patterns in the coronary arteries, aortic branches, abdominal visceral arteries, and terminal aorta [30]. Based on this, it can be postulated that VSMCs exhibit differential susceptibility to inflammatory factors depending on their cellular origin, thereby influencing the heterogeneous progression of CVDs across various vascular territories.

## 3. Phenotypes of VSMCs

**Figure 1:**
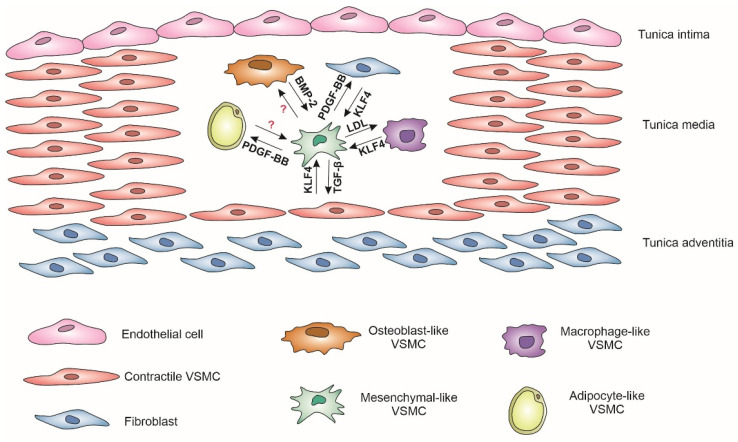
Factors regulating the phenotypic switching of VSMCs. BMP-2, bone morphogenetic protein 2; KLF4, Kruppel-like factor 4; LDL, low density lipo-protein; PDGF-BB, platelet-derived growth factor BB; TGF-β, transforming growth factor-β; VSMC, vascular smooth muscle cells.

Currently, there exists a hypothesis positing that the origin of vascular VSMCs and their phenotypic switching may impact the development of CVDs, although definitive evidence remains inconclusive. Under physiological conditions, contractile VSMCs secret a distinct profile of smooth muscle-specific markers, including alpha-actin 2 (ACTA2), myosin heavy chain 11 (MYH11), smoothelin, and other related proteins. However, during phenotypic switching, the expression levels of these markers are significantly downregulated. Accumulating evidence indicates that vascular VSMCs possess the capacity to undergo phenotypic transitions from a contractile state to alternative phenotypes, including the following: fibroblast-like, macrophage-like, secretory, osteogenic-like, and adipocyte-like [31]. To date, the precise molecular mechanisms underlying this phenotypic switching and its impact on CVD pathogenesis remain incompletely elucidated [32]. There are many factors regulating the phenotypic switching of VSMCs: TGF-β, Kruppel-like factor 4 (KLF4), various microRNAs (miRs), etc. (Figure 1). However, the exact mechanisms of switching VSMC phenotypes remain to be fully understood.

### 3.1. Contractile VSMCs

Normally, VSMCs exhibit a contractile phenotype and actively synthesize ECM components. Contractile VSMCs represent the predominant cell population within the normal human aortic wall. Contractile VSMCs exhibit an elongated fusiform morphology and express a characteristic panel of contractile smooth muscle markers, including the following: ACTA2, MYH11, smooth muscle protein 22-alpha (SM22α), smoothelin, and calponin 1 (CNN1). The expression of these proteins is regulated by transcription factors myocardin (MYOCD) and serum response factor (SRF), which collectively mediate the differentiation process into contractile VSMCs. The primary physiological roles of contractile VSMCs include the maintenance of vascular elasticity and provision of structural integrity to the blood vessel wall [33]. This multifaceted role is underpinned by their unique ability to dynamically regulate vessel diameter and maintain mechanical stability of the vasculature. Impairment of the contractile function of the VSMCs leads to the development of aneurysms and atherosclerosis. This may be attributed to the previously described phenomenon of switching VSMC phenotypes from contractile to fibroblast-like, macrophage-like, secretory, or synthetic in response to pathological stimulation [34]. Recent studies have demonstrated that the phenotypic modulation of VSMCs is involved in the pathogenesis of both aneurysms and atherosclerosis due to the remodeling of ECM and the accumulation of various types of differentiated VSMCs in atherosclerotic lesions [35].

### 3.2. Fibroblast-like VSMCs

Fibroblast-like VSMCs have the properties of both SMCs, such ascontractility and fibroblasts [36]. These cells exhibit heightened synthetic activity, actively secreting collagen and fibronectin, while demonstrating robust proliferative capacity. This phenotypic transition is characterized by the following: downregulation of contractile markers and upregulation of fibroblast-associated markers (lumican, biglycan and decorin). This fibroblast-like phenotype has been documented in experimental models of Marfan syndrome-associated aneurysms, providing valuable insights into the pathophysiological mechanisms underlying vascular remodeling in this condition [37]. Furthermore, recent research has demonstrated that fibroblast-like VSMCs exert a protective effect in the pathogenesis of coronary artery disease. This novel finding suggests a dual role of these cells in cardiovascular pathophysiology, balancing their potentially detrimental effects with beneficial contributions to vascular homeostasis [38].

### 3.3. Macrophage-like and Proliferating VSMCs

Recent experimental evidence has demonstrated that VSMCs can acquire a macrophage-like phenotype with phagocytic properties under the influence of cholesterol [39]. Macrophage-like VSMCs undergo significant molecular reprogramming, initiating robust expression of *Galectin-3* and classical macrophage-associated markers: CD11b, CD45, CD68, and CD116 [40]. Moreover, VSMCs isolated from aneurysm patients have been demonstrated to exhibit elevated phagocytic activity [41]. Also, Rong JX in his study showed that VSMCs, after absorbing cholesterol, turn into macrophage-like VSMCs and can then transform into foam cells [42].

### 3.4. Secretory and Synthetic Phenotypes of VSMCs

Secretory VSMCs are characterized by reduced expression of genes associated with contraction, and increased cell proliferation, migration, inflammation, and ECM synthesis. Secretory VSMCs do not upregulate cyclin and pro-inflammatory gene expression; synthetic VSMCs, on the contrary, participate in its activation and secrete pro-inflammatory cytokines. Phenotypic modulation from the contractile to secretory VSMC phenotype occurs in a number of aortic diseases, such as aneurysm and atherosclerosis of the aorta. But there is an opinion that secretory VSMCs can also play a protective role in CVDs, since they are associated with an increase in the thickness of the aortic wall [43].

### 3.5. Mesenchymal-like, Adipocyte-like, and Osteoblast-like VSMCs

Mesenchymal-like VSMCs are characterized by reduced expression of contractile proteins. They have a similar phenotype to mesenchymal cells and are able to differentiate into several cell lines. In one study, it was shown that VSMCs can acquire an adipocyte-like phenotype [44]. Adipocyte-like VSMCs were classified as beige adipocytes that regulate thermogenesis and have a low level of intercellular connections [45].

Osteoblast-like VSMCs are characterized by increased secretion of calcification markers: osteogenic transcription factors MSX2 (Msh Homeobox 2), Cbfa1 (core-binding factor α-1, also known as RUNX2) and Sp7/Osterix, as well as chondrogenic transcription factor SOX9. The VSMCs switching from a normal to an osteoblast phenotype can be caused by various factors, such as calcium phosphate deposition in the aortic wall, hypertension, osteoporosis, rheumatoid arthritis, and other diseases [46]. It is important to note that the main factor leading to the switch of the VSMCs phenotype from contractile to osteoblast-like is the protein BMP-2 (bone morphogenetic protein 2) [47]. BMP-2 enhances phosphate uptake in VSMCs, which is a key step in the calcification process, and induces the expression of genes associated with osteoblast differentiation, such as Runx2 [48]. Thus, BMP-2 promotes the transition of VSMCs from a contractile to an osteoblast-like phenotype, which can contribute to vascular calcification and potentially worsen conditions such as atherosclerosis and aneurysm [49].

## 4. Factors Regulating the Phenotypic Switching of VSMCs

### 4.1. TGF-β and KLF4

There are many factors regulating the phenotypic switching of VSMCs. Among these regulatory factors, TGF-β and KLF4 have been extensively studied due to their critical roles in regulating VSMCs phenotypic switching.

In 1993, research revealed that TGF-β1 is both synthesized and activated by neural crest cells [50]. Later, it was demonstrated that the influence of TGF-β1 on neural crest cells in vitro modulates both differentiation and mitogenesis [51] and also stimulates the synthesis of ECM proteins fibronectin and procollagen I [52], which are essential for neural crest cell migration. TGF-β is one of the most studied signaling pathways involved in vascular development and differentiation [53]. Mutations in genes associated with TGF-β (*TGF-β 1/2*, *SMAD2/3* or *TGFB2/3*) can lead to the formation of aortic aneurysms associated with hereditary connective tissue diseases, for example, Loes–Dietz syndrome and Marfan syndrome. TGF-β is also involved in the differentiation of VSMCs [54]. Disruption of TGF-β signaling during VSMC differentiation can induce a phenotypic switch from the normal VSMC phenotype to a mesenchymal-like state, resulting in VSMCs that can give rise to adipocytes, chondrocytes, osteoblasts, and macrophage-like cells. It is also worth noting that people with aortic aneurysm have a significant increase in TGF-β levels [55]. Nevertheless, the role of the TGF-β signaling pathway in aneurysm pathogenesis remains a subject of ongoing debate in the scientific community. TGF-β probably has several pathways and may play a different role in different cell types and at different stages of the disease.

It is well known that the different isoforms of TGF-β exhibit distinct biological activities. TGF-β1 is the most widely studied agent that possesses various effects on VSMCs leading to aneurysm development, including pro-fibrotic condition. In this regard TGF-β1 is considered as a promising therapeutic target in aneurysm treatment [56]. TGF-β2 activated by ECM protein thrombospondin-1 is involved in aneurism development, promoting VSMCs migration and proliferation [57]. At the same time, the effects of TGF-β3 on VSMCs are not well studied, but it was shown in several studies that TGF-β3 plays an important role in the synthesis of the ECM and has pro-regenerative potential in terms of fibroblasts that in turn can influence VSMC differentiation [58].

In a murine study, it was demonstrated that TGF-β can influence the phenotypic switching not only of VSMCs, but conversely, facilitate the transition of neural crest-derived stem cells to a VSMC-like phenotype [59]. In addition, in vitro treatment with TGF-β1 induces the expression of α-smooth muscle actin in ocular cells of neural crest origin, such as corneal keratocytes, corneal endothelial cells, and trabecular meshwork cells. Notably, the exposure of murine lens and cornea to elevated concentrations of TGF-β resulted in robust proliferation of corneal stromal mesenchymal cells, which concurrently exhibited active expression of α-smooth muscle actin. These findings collectively suggest that excessive TGF-β signaling may disrupt normal differentiation of corneal and lenticular cells while simultaneously triggering phenotypic switching in stromal mesenchymal cells.

Moreover, increased expression of TGF-β may contribute to the pathogenesis of asthma and chronic obstructive pulmonary disease, since TGF-β is a pleiotropic mediator involved in many biological functions of the lungs [60]. The TGF-β family of growth factors is crucial for the normal development of the lungs and regulates the processes of recovery and regeneration in the lungs of healthy adult [61]. TGF-β signaling in the lungs is strictly controlled, and abnormal expression of TGF-β can provoke phenotypic and functional changes in the airway SMCs (ASMCs), which can lead to the development of asthma and other lung diseases [62]. In some studies, it has been shown that transgenic overexpression of biologically active TGF-β1 causes structural disorders in the respiratory tract, parenchyma, and pulmonary vascular network, including excessive fibrosis [63,64].

Thus, disruption of the normal regulation of the TGF-β signaling pathway leads to SMC dysfunction, which is an important factor in the pathogenesis of various diseases, especially aortic aneurysms [65]. Understanding these mechanisms opens up prospects for the development of new approaches to the prevention and treatment of aneurysms aimed at correcting the imbalance of TGF-β-mediated signaling pathways.

Another factor that participates in the regulation of cell proliferation and plays a key role in the process of dedifferentiation of the normal VSMC phenotype into a mesenchymal-like phenotype is the KLF4 transcription factor [66]. KLF4 is regulated by various signaling complexes at the transcriptional and post-translational levels. Platelet-derived growth factor PDGRF-BB triggers increased expression of KLF4 using transcription factor Sp1 (specificity protein1), which inhibits the differentiation of VSMCs [67]. However, increased expression of KLF4 reduces the secretion of previously described contractile markers in VSMCs and induces the phenotypic modulation of VSMCs [68]. During dedifferentiation, VSMCs begin to actively express mesenchymal markers such as stem cell antigen-1 (SCA1), CD34, and CD44 [69].

Thus, TGF-β and KLF4 are the main factors leading to the phenotypic switching of SMCs, and as a result, the development of CVDs and other diseases.

### 4.2. The Impact of Immune System Cells on VSMC Phenotype Modulation

It is noteworthy that, in addition to transcriptional and molecular factors, phenotypic switching of VSMCs can be modulated by immune system cells, specifically through cytokines and chemokines.

It is well known that macrophages are the main immune cells that play an important role in the pathogenesis of atherosclerosis [70]. A recent study conducted on Yucatan microswine suggests that IL-6, IL-1b, and TNF-α secreted by macrophages may have a differential effect on the phenotype of VSMCs, resulting in switching of the VSMC phenotype from contractile to macrophage-like, which leads to the development of chronic inflammation in the vascular wall [71].

In addition to macrophages, CD8^+^ T cells significantly contribute to the development of the pathological phenotype in VSMCs. Schäfer S. and colleagues demonstrated, in a murine study, that CD8^+^ T cells contribute to both the destabilization of atherosclerotic plaques and the phenotype switching of VSMCs comprising the fibrous cap of atherosclerotic plaques from a contractile to a macrophage-like phenotype [72]. Thus, CD8^+^ T cells contribute to the dedifferentiation of VSMCs, suppress their proliferation and migration, and also contribute to the phenotypic modulation of VSMCs.

In addition, neutrophils can also affect the functioning of the VSMCs. In one of the studies, the authors demonstrated that VSMCs forming a fibrous capsule of atherosclerotic plaque can attract neutrophils, which actively secrete histone H4, which lyses VSMCs, resulting in unstable atherosclerotic plaque, that leads to its rupture [73].

### 4.3. miRs

In addition to the factors TGF-β and KLF4, miRs can also affect changes in the phenotype of VSMCs. miRs are non-coding RNAs with a length of 18–25 nucleotides, and their main function is to regulate genome expression, which in turn affects changes in the main signaling pathways and metabolism of cells [74]. Thus, miRs play a pivotal role in various cellular processes such as cellular differentiation, proliferation, and apoptosis, and their effect may depend on which cells they are expressed in (Table 1) [75].

Recent studies have shown that miR-143/145, miR-155, miR-21, and miR-126 contribute to vascular inflammation [86]. Moreover, miR-143/145, miR-221/222, miR-21, and miR-124 are involved in the phenotypic switching of VSMCs [87].

#### 4.3.1. miRs-143/145

miRs-143/145 have been demonstrated to exhibit abundant expression in the vascular wall and are recognized as both markers of the VSMC phenotype and regulatory modulators capable of controlling vascular lesion formation [88]. Also, miRs-143/145 regulate the development of vascular pathologies through MYOCD, TGF-β1, and KLF4. A recent study demonstrated that miR-145 is involved in the regulation of KLF4 expression, which subsequently activates the mechanisms of VSMC differentiation [89]. It was demonstrated that the inhibition of miR-145 resulted in increased KLF4 expression, which in turn led to decreased expression of contractile markers, particularly the myosin heavy chain, in VSMCs. This suggests that VSMCs may undergo a phenotypic transition from a contractile to a secretory state [90], potentially leading to airway remodeling and subsequently contributing to the development of various respiratory diseases, such as bronchial asthma. In a murine study, it was shown that a deficiency of miRs-143/145 led to a thinning of the vessel wall and a decrease in the number of contractile VSMCs. This confirms that miRs-143/145 play an important role in the process of phenotypic modulation of VSMCs and inhibition of the proliferation of VSMCs [91]. It is also worth noting that overexpression of miRs-143/145 triggers the switching of the smooth muscle cell phenotype from synthetic to contractile in aortic aneurysm and reduces the incidence of thoracic aortic aneurysm in mice [92]. This may indicate that miRs-143/145 expression levels are associated with the severity of CVDs.

#### 4.3.2. miRs-221/222

It is well established that miRs-221/222 exert dual regulatory effects on both VSMCs and endothelial cells. Consequently, these miRs play a crucial role in vascular remodeling processes that occur in response to vascular wall injury [93]. Studies have shown that VSMCs actively express miRs-221/222 [94]. However, a decrease in miRs-221/222 expression in VSMCs can lead to calcium deposition in the vessel wall and, as a result, a switch from the normal VSMC phenotype to the osteoblast-like phenotype [95]. It is also worth noting that in some studies, scientists have demonstrated that an increase in the expression of miRs-221/222 in VSMCs leads to a switch in their phenotype from contractile to synthetic, as a result of which VSMCs begin to actively express pro-inflammatory cytokines, which leads to local inflammation of the vessel wall [96]. Thus, miRs-221/222 can be a potential therapeutic target for the treatment of atherosclerosis and aneurysms.

#### 4.3.3. miR-21

miR-21 plays an important role in the pathogenesis of CVDs, such as atherosclerosis and aneurysm [97]. Thus, abnormal expression of miR-21 by VSMCs can lead to a switch in the phenotype of VSMCs from contractile to synthetic, which leads to the progression of atherosclerosis [98]. In addition, studies indicate that with the development of aortic aneurysm, miR-21 expression by VSMCs increases, but overexpression of miR-21, on the contrary, reduces apoptosis of VSMCs and inhibits the progression of aortic aneurysm [99]. Thus, miR-21 can be used as a diagnostic tool for screening patients with aortic aneurysm.

#### 4.3.4. miR-24

miR-24 is another microRNA that is involved in the development of CVDs. Scientists have shown that miR-24 plays an important role in the regulation of myoblast differentiation and can be inhibited by TGF-β [100]. As previously described, *SMAD3* can mediate the suppression of myogenesis of TGF-β. However, it is known that TGF-β can inhibit miR-24 only if it has a SMAD binding site in the promoter region [101]. Consequently, it can be hypothesized that TGF-β is unable to regulate miR-24 expression in SMAD3-deficient cells. Furthermore, patients with aneurysms often exhibit significant mutations in the *SMAD3* gene, which subsequently result in functional impairments of VSMCs [102]. Thus, it can be postulated that elevated miR-24 transcription in VSMCs may induce their differentiation into a pathological phenotype, thereby contributing to the development of CVDs.

#### 4.3.5. miR-126

At the moment, there is a hypothesis that reduced or increased expression of miR-126 may indicate the risk of developing CVDs [103]. A recent study has shown that miR-126 can act as a marker of endothelial cell dysfunction, which is one of the key causes in the development of CVDs [104]. Also, many studies suggest that although miR-126 is not expressed in VSMCs, it can participate in the regulation of their functions, which leads to a decrease in the proliferation and migration of VSMCs [105], which is an important pathogenetic factor in the development of vascular pathology. Thus, miR-126 emerges as a promising biomarker for assessing the risk of CVDs development and may serve as a valuable diagnostic tool in clinical practice.

#### 4.3.6. miR-155

Extensive research evidence indicates that miR-155 serves as a pivotal regulatory factor in the pathogenesis of CVDs, playing a critical role in their development and progression [106]. Nevertheless, the precise molecular mechanisms underlying this regulatory process remain to be elucidated. Researchers hypothesize that the cellular localization of miR-155 determines its functional impact, with variations in its expression levels potentially activating distinct molecular pathways involved in vascular wall inflammation. Specifically, the differential expression of miR-155 in various cell types may initiate divergent molecular responses contributing to inflammatory processes within the vasculature [107]. For example, increased expression of miR-155 in macrophages led to their transformation into foam cells and the development of atherosclerosis [108]. Another example is that a decrease in miR-155 expression in fibroblasts, on the contrary, may serve as a protective mechanism in heart fibrosis in diabetic patients [109]. Consequently, miR-155 can be a crucial biomarker not only for the development of CVDs but also represents a promising therapeutic target for their treatment.

## 5. The Development of miR-Based Therapeutic Strategies for the Treatment of CVDs

In summary, the aforementioned evidence underscores the potential utility of miRs as tools for early detection and prognostication of CVDs. Furthermore, miR-based therapeutics offer promising opportunities to specifically target genes involved in key pathological processes, including the following: lipid metabolism, inflammation, and the phenotypic modulation of VSMCs [110]. miR-based therapy has great prospects for the diagnosis and treatment of CVDs, as currently available protein-based biomarkers can give false results, resulting in incorrect diagnosis and treatment of patients [111].

Moreover, miR-based vaccines are also a promising area in the fight against CVDs. An example is the AtheroVax vaccine, which is aimed at treating chronic inflammation through modulation of the TNF-α receptor II splicing variant expression [112]. This vaccine might be an effective candidate for the treatment of atherosclerotic CVDs, as it is aimed at treating chronic inflammation that leads to the development of atherosclerosis. Scientists have already conducted preclinical studies of this vaccine in rats, during which they demonstrated that taking AtheroVax significantly reduced serum levels of TNF-α and IL-6, which may indicate a decrease in chronic inflammation [113].

Another example of a vaccine for the treatment of CVDs is VXX-401. This vaccine targets proprotein convertase subtilisin/kexin type 9 (PCSK9) inhibitors, which, in combination with statins, can significantly reduce LDL levels, thereby decreasing the risk of CVD development [114]. Scientists have already conducted studies on Javanese macaques, which demonstrated that VXX-401 is a highly immunogenic vaccine and reduces LDL levels by 30–40% without altering serum HDL levels. In vitro analyses have also shown that VXX-401 induces a safe humoral response and is well tolerated, without signs of autoimmunity or chronic inflammation [115]. These findings suggest that VXX-401 holds significant therapeutic potential for the treatment of CVDs.

Although miR-based therapeutics have demonstrated promising efficacy in preclinical studies, several challenges must be addressed to overcome potential limitations in their clinical application.

Thus, for the large-scale application of these vaccines in humans, it is important to identify the target genes and signaling pathways responsible for their cardiovascular effects in order to develop new therapeutic agents based on miRs. In addition, most preclinical miR studies have focused on site-specific phenotypic effects that may ignore non-target effects in other tissues. Thus, research is needed to focus on the effects of miRs with a systemic approach instead of site-specific approaches [116].

Another important task is to determine the appropriate translational dosing regimens in order to obtain minimal doses with maximum effectiveness and minimal side effects [117].

In addition, the high cost of developing and implementing new miR-based vaccines for the treatment of CVDs is another significant obstacle, as expensive production methods are used to create these vaccines. This can limit accessibility, especially in low- and middle-income countries. Addressing these cost issues requires coordinated efforts to reduce production costs, increase funding for innovative treatments, and ensure equal access for all patients, regardless of economic status [118].

## 6. Conclusions

The studies reviewed herein demonstrate the pivotal role of VSMCs in the pathogenesis of CVDs. It has been established that both the origin of VSMCs and their phenotypic switching represent critical factors in the development of vascular disorders. The mechanisms underlying CVD pathogenesis are governed by a complex network of signaling pathways that regulate VSMC behavior. The phenotypic plasticity of these cells and their capacity to transition to pathological phenotypes play a crucial role in the development of vascular pathology.

Moreover, current evidence indicates that miRs exert a significant impact on VSMCs, modulating their functional characteristics and contributing to the pathogenesis of CVDs. The investigation of miRs holds particular significance for the development of innovative therapies based on targeted effects on miRs and signaling pathways that regulate VSMC behavior. Strategies focused on studying miR expression appear to be the most promising in the context of CVDs treatment due to their capacity to influence key mechanisms of disease development.

Thus, a comprehensive study of the origin of VSMCs, the mechanisms governing their phenotypic switching, and the role of miRs in regulating their functions represents a promising area in cardiology, thereby opening new opportunities for combating CVDs.

## Figures and Tables

**Table 1:** Localization of miRs in cells.

miR	Localization
miRs-143/145	vascular smooth muscle cells [76]
miRs-221/222	vascular smooth muscle cells [77]
miR-21	vascular smooth muscle cells [78], macrophages [79]
miR-24	vascular smooth muscle cells [80], endothelial cells [81]
miR-126	endothelial cells [82]
miR-155	macrophages [83], fibroblasts [84], endothelial cells [85]

## Abbreviations

ACTA2	Alpha-actin 2
ASMCs	Airway smooth muscle cells
BMP2	Bone morphogenetic protein 2
CNN1	Calponin 1
CVDs	Cardiovascular diseases
ECM	Extracellular matrix
IL	Interleukin
KLF4	Kruppel-like factor 4
LDL	Low density lipoprotein
miR	MicroRNA
MYH11	Myosin heavy chain 11
MYOCD	Myocardin
PDGRF-BB	Platelet-derived growth factor BB
SHF	Second heart field
SN22α	Smooth muscle protein 22-α
SRF	Serum response factor
TGF-β	Transforming growth factor-β
TNF	Tumor necrosis factor
VSMCs	Vascular smooth muscle cells
